# 599. Early Psychotropic Intervention and Clinical Outcomes in Pediatric Burn Survivors: A Retrospective Cohort Study

**DOI:** 10.1093/jbcr/irag033.403

**Published:** 2026-04-06

**Authors:** Eric W Lian, Young Jo, Minju Hwang, Tahmid Hussain, Whitman Wiggins, Md Mahmudul Hasan

**Affiliations:** University of Florida Health Shands Burn Center, Gainesville, FL; University of Florida, Gainesville, FL; University of Florida, Gainesville, FL; University of Florida, Gainesville, FL; Department of Surgery, University of Florida, Gainesville, FL; University of Florida, Gainesville, FL

## Abstract

**Introduction:**

Burn injuries in children are not only physically devastating but also associated with significant psychiatric comorbidities. This study seeks to identify whether early treatment with psychotropics decrease post-discharge development of psychiatric illness and surgical complications among pediatric patients hospitalized after burn injuries.

**Methods:**

This is a retrospective cohort study using MarketScan Commercial Claims and Encounters Database from 2011 to 2023. The cohort included first-time inpatient burn admissions from January 2011 to June 2023, excluding patients with psychiatric diagnoses in the year prior to admission. Intervention is defined as psychotropics being filled within seven days of discharge. Outcomes are defined as presence of new psychiatric diagnosis or surgical complications per International Classification of Diseases 9 and 10 codes within the six month follow-up period.

**Results:**

The cohort included 770 patients (139 intervention, 631 control) aged 6-17. The populations had similar baseline characteristics. Antihistamines was the most prescribed medication class, with 38 patients in the intervention group and 33 patients in the control group. SSRIs were the second most prescribed class, with 30 patients in the intervention group and 32 patients in the control group. The intervention group had higher odds of a new mental health diagnosis (OR = 1.844, 95% CI:1.199-2.836, *p*=.0053) and post-operative complications (OR = 2.042, 95% CI: 1.353-3.079, *p*=.0007) versus controls. Increasing age was associated with higher odds of new psychiatric diagnosis (OR = 1.138 per year, *p*<.0001), and third degree burns were associated with increased post-operative complications (OR = 2.476, *p*=.0006). There was no statistically significant difference between male and female populations.

**Conclusions:**

Pediatric burn patients who received psychotropic medications during initial hospitalization are at higher risk for post-operative complications and psychiatric comorbidities than those who do not. The limitations of the database and the small number of patients in each psychotropic category limited this study. More data is needed to clarify the relationship and to determine if early psychotropic treatment helps decrease incidence of poor outcomes.

**Applicability of Research to Practice:**

Presence of psychotropics alone does not appear to be enough to decrease risk of psychiatric or surgical complications after hospitalizations for burn injuries among pediatric patients.

**Funding for the study:**

N/A.

